# Evaluation of [^64^Cu]Cu-NOTA-PEG_7_-H-Tz for Pretargeted Imaging in LS174T Xenografts—Comparison to [^111^In]In-DOTA-PEG_11_-BisPy-Tz

**DOI:** 10.3390/molecules26030544

**Published:** 2021-01-21

**Authors:** Christian B. M. Poulie, Jesper T. Jørgensen, Vladimir Shalgunov, Georgios Kougioumtzoglou, Troels Elmer Jeppesen, Andreas Kjaer, Matthias M. Herth

**Affiliations:** 1 Department of Drug Design and Pharmacology, Faculty of Health and Medical Sciences, University of Copenhagen, Jagtvej 160, 2100 Copenhagen, Denmark; christian.poulie@sund.ku.dk (C.B.M.P.); vladimir.shalgunov@sund.ku.dk (V.S.); dkr724@alumni.ku.dk (G.K.); 2 Department of Clinical Physiology, Nuclear Medicine & PET, Rigshospitalet, Blegdamsvej 9, 2100 Copenhagen, Denmark; jespertj@sund.ku.dk (J.T.J.); troelsej@sund.ku.dk (T.E.J.); 3 Cluster for Molecular Imaging, Department of Biomedical Sciences, University of Copenhagen, Blegdamsvej 3, 2200 Copenhagen, Denmark

**Keywords:** tetrazine, pretargeted imaging, radiochemistry, PET, SPECT, cancer

## Abstract

Pretargeted nuclear imaging for the diagnosis of various cancers is an emerging and fast developing field. The tetrazine ligation is currently considered the most promising reaction in this respect. Monoclonal antibodies are often the preferred choice as pretargeting vector due to their outstanding targeting properties. In this work, we evaluated the performance of [^64^Cu]Cu-NOTA-PEG_7_-H-Tz using a setup we previously used for [^111^In]In-DOTA-PEG_11_-BisPy-Tz, thereby allowing for comparison of the performance of these two promising pretargeting imaging agents. The evaluation included a comparison of the physicochemical properties of the compounds and their performance in an ex vivo blocking assay. Finally, [^64^Cu]Cu-NOTA-PEG_7_-H-Tz was evaluated in a pretargeted imaging study and compared to [^111^In]In-DOTA-PEG_11_-BisPy-Tz. Despite minor differences, this study indicated that both evaluated tetrazines are equally suited for pretargeted imaging.

## 1. Introduction

Pretargeted imaging has attracted increased interest over the last decades and is an emerging and fast developing field within oncology [1]. Until recently, pretargeted strategies were mainly based on the noncovalent interactions between the binding of biotin to (strept)avidin, duplex formation of two complementary strands of oligonucleotides or via the use of bispecific antibodies [2]. More recently, the focus has shifted to covalent bond forming methods [3,4,5]. This requires reactions that are not only bioorthogonal, but also exceptionally fast for in vivo applications. Several different methods exist, but the tetrazine ligation between a *trans*-cyclooctene (TCO) and a tetrazine (Tz) is currently considered the most promising reaction for this purpose [6]. The reaction is initiated via an inverse-electron-demand Diels–Alder (IEDDA) reaction and undergoes subsequently a retro-Diels-Alder reaction, with the expulsion of nitrogen gas. As such, the tetrazine ligation is irreversible [7]. Usually, monoclonal antibodies (mAbs), but also other nanomedicines, are used as pretargeting vectors. They possess exceptional targeting abilities [8]. Typically, TCOs are conjugated to the pretargeting vector and the tetrazine is used as the imaging probe [1,9].

[^111^In]In-DOTA-PEG_11_-BisPy-Tz (**2**), was the first radiolabeled Tz to be applied for this purpose, using TCO conjugated CC49 (CC49-TCO), an mAb that targets tumor associated glycoprotein 72 (TAG-72), as the pretargeting vector [4]. The TAG-72 antigen has been shown to have limited internalization when CC49 binds, making it ideal for pretargeting approaches [10]. Using this setup, [^111^In]In-DOTA-PEG_11_-BisPy-Tz (**2**) showed high tumor uptake, as well as high tumor-to-background ratios, in mice bearing human colon carcinoma xenografts of LS174T cancer cells that overexpress TAG72. Many groups have continuously worked to improve pretargeting strategies based on the tetrazine ligation. For example, we have recently used the same experimental setup to compare the performance of [^111^In]In-DOTA-PEG_11_-BisPy-Tz (**2**) to a ^68^Ga-labeled variant of the tracer [11]. In general, it is usually difficult to compare the performance of different tetrazine-based molecular imaging probes, because factors such as animal strain, tumor models, pretargeting vectors, and evaluation time points are varied [12,13,14,15,16,17,18,19,20]. This is also the case for [^64^Cu]Cu-NOTA-PEG_7_-H-Tz (**1**), another radiolabeled tetrazine, which is reported to be highly effective for pretargeted imaging, however, using a different TCO-conjugated pretargeting vector and mouse tumor model [3]. In order to allow for direct comparison of tetrazine-based imaging agents, there is a need to harmonize the experimental setup.

**Figure 1:**
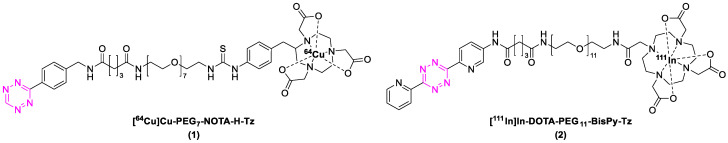
The chemical structures of [^64^Cu]Cu-NOTA-PEG_7_-H-Tz (**1**) and [^111^In]In-DOTA-PEG_11_-BisPy-Tz (**2**).

In this study, we evaluated [^64^Cu]Cu-NOTA-PEG_7_-H-Tz (**1**) in mice bearing LS174T tumor xenografts, using CC49-TCO as a pretargeting vector. The same setup that was previously used for the evaluation of [^111^In]In-DOTA-PEG_11_-BisPy-Tz (**2**) by us and others [3,4,11], and allows us to compare the in vivo performance of [^64^Cu]Cu-NOTA-PEG_7_-H-Tz to previous results [11]. Figure 1 displays both tetrazines used in this study. There are some differences between positron emission tomography (PET) used for imaging of [^64^Cu]Cu-NOTA-PEG_7_-H-Tz (**1**), and single-photon emission computed tomography (SPECT) used for imaging of [^111^In]In-DOTA-PEG_11_-BisPy-Tz, for example with regard to the resolution and sensitivity [21]. In general, these differences are less pronounced for preclinical systems, and will as such not be included in this comparison. However, it could affect the performance if translated to clinical settings, and should therefore also be taken into consideration [22]. 

## 2. Results and Discussion

### 2.1. Comparison of Physicochemical Properties and In Vitro Stability

The structural design of [^64^Cu]Cu-NOTA-PEG_7_-H-Tz (**1**) and [^111^In]In-DOTA-PEG_11_-BisPy-Tz (**2**) follows the same principle. Both tetrazines consist of a reactive tetrazine moiety and a polar chelator, connected via a polyethylene glycol (PEG) spacer. However, the overall charge of the metal complex is −1 for the copper (Cu^II^)-NOTA complex (**1**) and 0 for the indium (In^III^)-DOTA complex (**2**). The lipophilicity of the compounds is also fairly distinct. Whereas the [^64^Cu]Cu-NOTA-PEG_7_-H-Tz (**1**) possesses a log*D*_7.4_ of approximately −2.44, the [^111^In]In-DOTA-PEG_11_-BisPy-Tz (**2**) displays a log*D*_7.4_ of ca. −4.51 [3,23]. Moreover, the H-Tz in **1** displays a slightly lower reactivity than the BisPy-Tz in **2** (Table 1). We recently showed that higher polarity and higher reactivity of tetrazines are beneficial to achieve higher target-to-background ratios [24]. As such, the [^111^In]In-DOTA-PEG_11_-BisPy-Tz (**2**) may have better imaging properties. However, only trends and not absolute relationships were identified within our study, and target-to-background ratios were almost identical at a cLogD_7.4_ of approximately <−3 and a rate constant > 200 M^−1^s^−1^ almost identical [24]. Both tetrazines studied herein have values in these ranges. Therefore, we cannot predict whether the faster reaction kinetics and the lower lipophilicity of [^111^In]In-DOTA-PEG_11_-BisPy-Tz (**2**) may, in fact, turn out to be of greater benefit for pretargeted imaging. Both probes revealed similar in vitro stabilities, in PBS, as well as in serum [3,4]. After 2 h at 37 °C, approximately 85% of the compounds were intact. Table 1 lists these relevant physicochemical properties, the reactivity, and the in vitro stability data of both compounds. 

### 2.2. Ex Vivo Blocking Study

**Figure 2:**
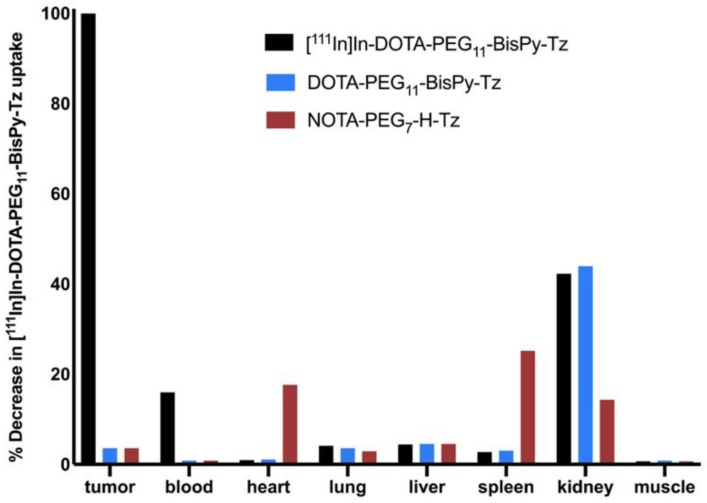
Blocking study of NOTA-PEG_7_-H-Tz and DOTA-PEG_11_-BisPy-Tz. The tissue uptakes are normalized to the average tumor uptake of [^111^In]In-DOTA-PEG_11_-BisPy-Tz (**2**). Tumor-bearing mice (*n* = 3/group) were injected with CC49-TCO, 72 h before administration of the unlabeled tetrazines. After one hour, [^111^In]In-DOTA-PEG_11_-BisPy-Tz (**2**) was administered. Ex vivo biodistribution were performed 22 h post [^111^In]In-DOTA-PEG_11_-BisPy-Tz (**2**) injection.

NOTA-PEG_7_-H-Tz was first evaluated in an in-house-developed ex vivo blocking assay [24]. For direct comparison, DOTA-PEG_11_-BisPy-Tz was also included in the test. The principle behind this assay is based on traditional receptor competition studies. In short, 72 h prior to the injection of the Tz, CC49-TCO (100 μg, ~7 TCOs/mAb, 3.9 nmol TCO) was administered into BALB/c nude mice, bearing LS174T colon carcinoma xenografts. Unlabeled Tz (10 equivalents with respect to the injected quantity of TCO) were administered 1 h before administration of [^111^In]In-DOTA-PEG_11_-BisPy-Tz (**2**) (1 equivalent with respect to injected TCO amount). Animals were euthanized after 22 h, tissues dissected, and the ex vivo biodistribution of [^111^In]In-DOTA-PEG_11_-BisPy-Tz (**2**) determined using a gamma counter. This allowed for quantification of the blocking effect of the unlabeled tetrazines. Both compounds were able to block the tumor uptake by >96% (Figure 2). Minor differences in uptake of [^111^In]In-DOTA-PEG_11_-BisPy-Tz (**2**) was found between animals pretreated with NOTA-PEG_7_-H-Tz and DOTA-PEG_11_-BisPy-Tz in heart, spleen, and kidney tissue. 

### 2.3. Pretargeted Imaging

**Figure 3:**
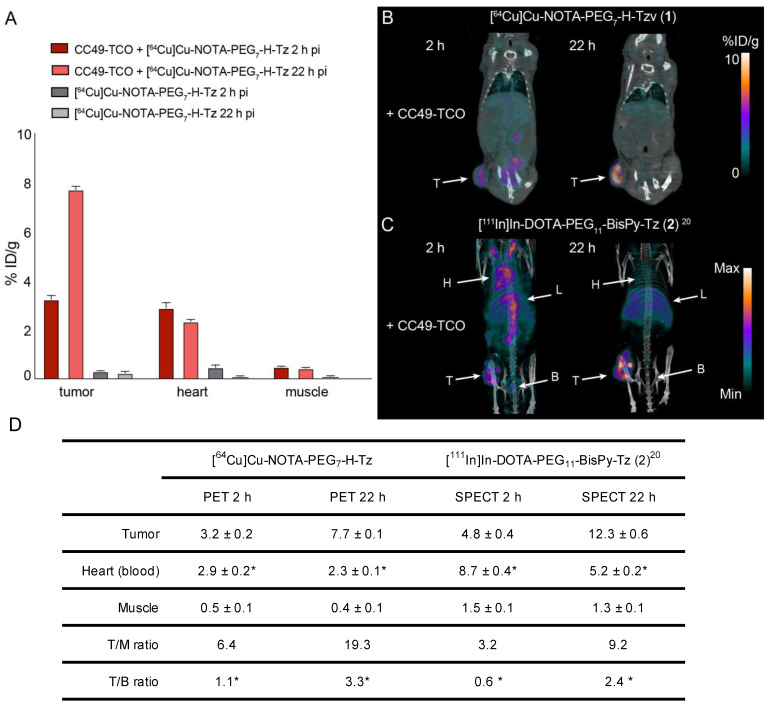
Pretargeted PET/CT imaging. (**A**) PET image-derived mean tissue uptake values in % ID/g (mean ± SEM, *n* = 4). (**B**) PET images of [^64^Cu]Cu-NOTA-PEG_7_-H-Tz (**1**) (in mice bearing colon carcinoma LS174T xenografts). The mice were pretreated with CC49-TCO 72 h prior to injection of [^64^Cu]Cu-NOTA-PEG_7_-H-Tz (**1**). Imaging was performed 2 and 22 h post injection. T: Tumor. (**C**) Data adopted from reference [11]. Maximum intensity projections from SPECT/CT scans of [^111^In]In-DOTA-PEG_11_-BisPy-Tz (**2**) in mice bearing colon carcinoma LS174T xenografts and pretreated with CC49-TCO 72 h prior to injection of the tracer. T: tumor, B: bladder, L: lungs, H: heart. (**D**) Image-derived mean uptake values (% ID/g), tumor-to-muscle (T/M) ratios and tumor-to-blood (T/B) ratios from [^64^Cu]Cu-NOTA-PEG_7_-H-Tz (**1**) PET scans at 2 h and 22 h p.i. in nude BALB/c mice bearing subcutaneous LS174T tumor xenografts pretreated with CC40-TCO (*n* = 4). For comparison, data from [^111^In]In-DOTA-PEG_11_-BisPy-Tz (**2**) previously obtained are also included (*n* =3).^20^ Data are shown as mean ± standard error of mean (SEM). * Image-derived uptake in heart from SPECT and PET images used as a surrogate for blood.

Pretargeted imaging was performed using a similar setup to the ex vivo blocking assay. CC49-TCO was administered 72 h prior to the administration of the radiolabeled tetrazine and PET imaging of [^64^Cu]Cu-NOTA-PEG_7_-H-Tz (**1**) was carried out 2 and 22 h after the radioligand administration. The tumors were clearly visible at both time points and the background uptake was generally low (Figure 3B). Image analysis showed that the tumor uptake increased from 3.2 ± 0.3 percentage of injected dose per gram (% ID/g, mean ± SEM) 2 h post injection (p.i.) to 7.7 ± 0.2% ID/g 22 h p.i. (Figure 3A,D). The corresponding tumor-to-muscle (T/M) ratios were 6.4 and 19.3, respectively. No specific tumor uptake was detected in control animals that were injected with [^64^Cu]Cu-NOTA-PEG_7_-H-Tz (**1**) without any prior administration of CC49-TCO.

The uptake in tumor tissue as well as in other organs of [^64^Cu]Cu-NOTA-PEG_7_-H-Tz (**1**) was slightly lower than those previously found when evaluating [^111^In]In-DOTA-PEG_11_-BisPy-Tz (**2**), using the same setup (Figure 3C,D) [11]. Significant amounts of activity of 2.9 ± 0.5% ID/g were also found in the heart (surrogate for the blood activity) after 2 h, which slightly decreased over the next 20 h to 2.3 ± 0.1% ID/g, resulting in tumor-to-blood (T/B) ratios of 1.1 and 3.3, respectively (Figure 3D). These values are significantly lower than those observed for [^111^In]In-DOTA-PEG_11_-BisPy-Tz (**2**), and indicate that [^64^Cu]Cu-NOTA-PEG_7_-H-Tz (**1**) is excreted at a faster rate [11]. This also results in higher tumor-to-background ratios (Figure 3D). However, free Tz is excreted within the first hour and as such, the observed blood uptake cannot be explained by free circulating Tz. A likely explanation for the unexpected high blood uptake over 22 h is that a fraction of the radiolabeled Tz ligates to residual CC49-TCO still circulating within the blood stream. This can also explain the observed increase in tumor uptake over time, as the ligation adduct in the circulation gradually accumulates at the target side over time. This phenomenon has also previously been described for [^111^In]In-DOTA-PEG_11_-BisPy-Tz (**2**) [5].

In conclusion, we evaluated the performance of [^64^Cu]Cu-NOTA-PEG_7_-H-Tz (**1**) in vivo using a setup previously used for [^111^In]In-DOTA-PEG_11_-BisPy-Tz (**2**) and were therefore able to directly compare the performance of the two imaging agents. Both radiolabeled tetrazines performed equally well in an ex vivo blocking assay. Similarly, the pretargeted imaging study revealed only minor differences in performance between the two imaging agents. Although the tumor uptake of [^64^Cu]Cu-NOTA-PEG_7_-H-Tz (**1**) was lower than that of [^111^In]In-DOTA-PEG_11_-BisPy-Tz (**2**), the former cleared at a higher rate, resulting in higher T/B and T/M ratios. For both radiolabeled tetrazines, however, the tumors were easily detected on the images, as early as 2 h p.i. Based on this, we believe that [^64^Cu]Cu-NOTA-PEG_7_-H-Tz (**1**) and [^111^In]In-DOTA-PEG_11_-BisPy-Tz (**2**) are equally suited for a pretargeted approach.

## 3. Experimental Section

### 3.1. Organic Chemistry

All reactions involving dry solvents or sensitive agents were performed under a nitrogen atmosphere and glasswares were dried prior to use. Commercially available chemicals were used without further purification. Solvents were dried prior to use with an SG water solvent purification system (Pure Process Technology, Nashua, NH, USA) or dried by standard procedures, and reactions were monitored by analytical thin-layer chromatography (TLC, Merck silica gel 60 F_254_ aluminum sheets). Flash chromatography was carried out using Merck silica gel 60Å (35–70 μm) (Sigma-Aldrich, Darmstadt, Germany). The ^1^H-NMR spectra were recorded on a 400 MHz Avance III or 600 MHz Avance III HD, and ^13^C NMR spectra on a 101 MHz Avance III or 151 MHz Avance III HD (Bruker, Bremen, Germany). Analytical HPLC was performed using an UltiMate HPLC system consisting of an LPG-3400A pump (1 mL/min), a WPS-3000SL autosampler, and a 3000 Diode Array Detector installed with a Gemini-NX C18 (250 × 4.60 mm, 3 μm) column. Solvent A: H_2_O + 0.1% TFA; Solvent B: MeCN-H_2_O 9:1 + 0.1% TFA. For HPLC control, data collection, and data handling, Chromeleon software v. 6.80 was used. Preparative HPLC was carried out on an Ultimate HPLC system with an LPG-3200BX pump, a Rheodyne 9721i injector, a 10 mL loop, an MWD-3000SD detector (200, 210, 225 and 254 nm) (ThermoScientific, Loughborough, UK), and a Gemini-NX C18 (250 × 21.2 mm, 5 μm) column for preparative purifications, or a Gemini-NX C18 (250 × 10.00 mm, 5 μm) column for semipreparative purifications. Solvent A: H_2_O + 0.1% TFA; Solvent B: MeCN-H_2_O 9:1 + 0.1% TFA. For HPLC control, data collection, and data handling, Chromeleon software v. 6.80 was used. Chiral preparative HPLC was performed using the same instrumentation as mentioned above. UPLC-MS spectra were recorded using an Acquity UPLC H-Class series solvent delivery system equipped with an autoinjector coupled to an Acquity QDa and TUV detectors installed with an Acquity UPLC^®^BECH C18 (50 × 2.1 mm, 1.7 μm) column (Waters, Eschborn, Germany). Solvent A: 5% aq MeCN + 0.1% HCO_2_H; Solvent B: MeCN + 0.1% HCO_2_H. Usually, gradient ratios from A:B 1:0 to 1:1 (5 min) were performed depending on the polarity of the compounds. For data collection and data handling, MassLynx software (Waters, Eschborn, Germany) was used. Optical rotations were determined in a thermostated cuvette on an Anton Paar MCP300 Modular Circular Polarimeter. Compounds were dried under high vacuum or freeze dried using a CoolSafe Freeze Dryer (ScanVac, Lillerød, Denmark). The purity of compounds submitted for pharmacological characterization was determined by ^1^H-NMR and HPLC to be > 95%, unless otherwise noted.

2,2′,2″-(2-(4-(3-(1-(4-(1,2,4,5-tetrazin-3-yl)phenyl)-3,7-dioxo-11,14,17,20,23,26,29-heptaoxa-2,8-diazahentriacontan-31-yl)thioureido)benzyl)-1,4,7-triazonane-1,4,7-triyl)triacetic acid (NOTA-PEG_7_-H-Tz). NOTA-PEG_7_-H-Tz was synthesized as previously described by Zeglis et al. [3], starting from 2,5-dioxopyrrolidin-1-yl 5-((4-(1,2,4,5-tetrazin-3-yl)benzyl)amino)-5-oxopentanoate, which was synthesized as previously described by Nichols et al. [25], starting from *tert*-butyl (4-(1,2,4,5-tetrazin-3-yl)benzyl)carbamate in six overall steps. MS (ESI) *m/z* =1102.1 [M + H]^+^, 551.7 [M + 2H]^2+^; ^1^H NMR (600 MHz, MeOD) *δ* 10.35 (s, 1H), 8.58 (d, *J* = 8.3 Hz, 2H), 7.59 (d, *J* = 8.3 Hz, 2H), 7.36 (d, *J* = 8.2 Hz, 2H), 7.28 (d, *J* = 6.6 Hz, 2H), 4.53 (s, 2H), 3.81–3.59 (m, 42H), 3.56 (t, *J* = 5.5 Hz, 2H), 3.39 (t, *J* = 5.5 Hz, 3H), 3.24–3.13 (m, 2H), 2.96 (dd, *J* = 13.7, 6.9 Hz, 1H), 2.77 (dd, *J* = 14.8, 4.4 Hz, 1H), 2.36 (t, *J* = 7.5 Hz, 2H), 2.30 (t, *J* = 7.4 Hz, 2H), 1.98 (p, *J* = 7.5 Hz, 2H); ^13^C-NMR (151 MHz, MeOD) *δ* 182.3, 175.4, 168.8, 167.6, 159.30, 159.28, 145.7, 136.9, 132.3, 131.0, 129.43, 129.38, 125.6, 71.56, 71.54, 71.52, 71.51, 71.49, 71.27, 71.24, 70.6, 45.5, 43.8, 40.4, 36.2, 36.2, 23.2.

2,2′,2″-(10-(2,40,44-trioxo-44-((6-(6-(pyridin-2-yl)-1,2,4,5-tetrazin-3-yl)pyridin-3-yl)amino)-6,9,12,15,18,21,24,27,30,33,36-undecaoxa-3,39-diazatetratetracontyl)-1,4,7,10-tetraazacyclododecane-1,4,7-triyl)triacetic acid (DOTA-PEG_11_-BisPy-Tz). DOTA-PEG_11_-BisPy-Tz was synthesized as previously described by Rossin et al. [4], starting from 5-amino-2-cyanopyridine and cyanopyridine in six steps. MS (ESI) *m/z* = 640.0 [M + 2H]^2+^, 426.9 [M + 3H]^3+^; ^1^H-NMR (600 MHz, MeOD) *δ* 9.08 (d, *J* = 2.5 Hz, 1H), 8.89 (dd, *J* = 4.8, 0.9 Hz, 1H), 8.80–8.74 (m, 2H), 8.49 (dd, *J* = 8.7, 2.5 Hz, 1H), 8.18 (td, *J* = 7.8, 1.7 Hz, 1H), 7.74 (ddd, *J* = 7.6, 4.8, 1.2 Hz, 1H), 3.88–3.76 (m, 9H), 3.67–3.59 (m, 53H), 3.56 (q, *J* = 5.8 Hz, 5H), 3.39 (td, *J* = 5.5, 3.0 Hz, 5H), 2.55 (t, *J* = 7.3 Hz, 2H), 2.34 (t, *J* = 7.3 Hz, 2H), 2.04 (p, *J* = 7.4 Hz, 2H); ^13^C NMR (151 MHz, MeOD) *δ* 175.4, 174.3, 164.6, 164.5, 162.2, 151.4, 151.1, 145.2, 142.7, 140.3, 139.6, 128.4, 128.3, 126.2, 125.6, 117.8, 71.56, 71.53, 71.52, 71.50, 71.49, 71.47, 71.45, 71.44, 71.2, 71.07, 70.5, 70.2, 40.3, 36.8, 36.02, 22.5.

### 3.2. Radiochemistry

Reagents and solvents were obtained from suppliers and used without further purification. Metal-free water was used to make all of the standard solutions, and metal-free water was used for all experiments unless otherwise stated. Indium-111 (III) chloride was purchased from Curium (Oegstgeest, Netherlands) and copper-64 (II) chloride was purchased from Risø (Roskilde, Denmark). The radiolabeling for DOTA-PEG11-BisPy-Tz and H-Tz-PEG_7_-NOTA were performed as previously reported by Rossin et al. and Zeglis et al., respectively [3,4]. Analytical HPLC was performed on a Dionex system connected to a P680A pump, a UVD 170U detector, and a Scansys radiodetector. The system was controlled by Chromeleon 6.8 or Chromeleon 7.2 software.

[^64^Cu]Cu-NOTA-PEG_7_-H-Tz (**1**). A solution of [^64^Cu]CuCl_2_ in 0.1M HCl (230 µL, 211 MBq) was mixed with ammonium acetate buffer (1 M, pH 5, 30 μL) and NOTA-PEG_7_-H-Tz (23 µL of 2 mg/mL solution in water). The resulting mixture (pH 4) was placed on an agitating mixer and shaken at 300 rpm for 30 min at room temperature. HPLC showed < 10% unchelated copper in the solution. Then, the solution was diluted with water (2 mL) and passed through the Sep-Pak C18 Plus Light cartridge (Waters Cooperation, Milford, MA, USA) (rinsed with 10 mL EtOH first, then with 10 mL H_2_O before use). The cartridge was rinsed with water (2 mL), then with 10% MeOH in water (1 mL), then eluted with MeOH (0.6 mL). Flow direction used for the elution was opposite to the flow direction used for trapping crude [^64^Cu]Cu-NOTA-PEG_7_-H-Tz (**1**) and rinsing. The eluate was evaporated to dryness at 60 °C under nitrogen flow and the residue was redissolved in 0.9% saline (2 mL). [^64^Cu]Cu-NOTA-PEG_7_-H-Tz (**1**) was obtained in 78% isolated RCY with the RCP of 75%. Total tetrazine concentration was adjusted to 33 nmol/mL by adding unlabeled NOTA-PEG_7_-H-Tz solution. [^64^Cu]Cu-NOTA-PEG_7_-H-Tz (**1**) was obtained in 78% isolated RCY with the RCP of 75%. The molar activity of the [^64^Cu]Cu-NOTA-PEG_7_-H-Tz (**1**) solution used for the biodistribution studies and imaging experiments was 3 MBq/nmol. 

[^111^In]In-DOTA-PEG_11_-BisPy-Tz (**2**). [^111^In]In-DOTA-PEG_11_-BisPy-Tz (**2**) was synthesized as previously described by Edem et al. [11].

### 3.3. Animal Model

LS174T human colon cancer cells (ATCC, Manassas, VA, USA) was cultured in minimum essential medium supplemented with 10% fetal bovine serum, 1% L-glutamine, 1% sodium pyruvate, 1% non-essential amino acids, and 1% penicillin-streptomycin. Five week old female Balb/c nude mice (Charles River Laboratories, Cologne, Germany) were upon arrival, allowed to acclimatize for one week, and had at all times access to water and chow ad libitum. Xenografts were established by subcutaneous injection of 5 × 10^6^ cells suspended in 100 μL PBS in the left flank. The tumor size was monitored using caliper measurements (tumor volume = ½(length × width^2^).

### 3.4. Ex Vivo Blocking Assay

Mice bearing LS174T tumor xenografts were divided into groups based on their tumor volume (tumor volumes of ~100–300 mm^3^, n = 3 in each group) and were administered CC49-TCO antibodies (100 μg/100 μL, ~7 TCO/mAb, 3.9 nmol TCO). After 3 days, the animals were injected with nonradioactive DOTA-PEG_11_-BisPy-Tz (39 nmol/100 μL) or H-Tz-PEG_7_-NOTA (39 nmol/100 μL). One hour later, they were administered with [^111^In]In-DOTA-PEG_11_-BisPy-Tz (**2**) (~5 MBq/100 μL, 3.9 nmol) via the tail vein. The mice were euthanized after 22 h and tumor, blood, heart, lung, liver, spleen, kidney, and muscle tissue were collected. All samples were weighed and the radioactivity measured in a gamma counter (Wizard^2^, Perkin Elmer). Data were corrected for decay, tissue weight, and injected amount of radioactivity. Control animals exclusively receiving [^111^In]In-DOTA-PEG_11_-BisPy-Tz (**2**) were also included. The mean tumor uptake from the control group was used for normalization of all other uptake values, including from animals in the other groups. 

### 3.5. Pretargeted Imaging

Tumor-bearing mice were administered intravenously 100 µg of CC49-TCO 3 days prior to the imaging experiment. At the imaging experiment, mice were injected with [^64^Cu]Cu-NOTA-PEG_7_-H-Tz (**1**) (10 MBq/100 μL, 3.2 nmol) via the tail vein. Two hours after the injection, the animals were moved to a small animal PET/CT scanner (Inveon^®^, Siemens Medical Solutions, Malvern, PA, USA), and a PET acquisition (energy window of 350–650 KeV and a time resolution of 6 ns) was performed, followed by a CT scan (360 projections, 65 kV, 500 μA and 400 ms). This procedure was repeated at 22 h p.i. Sinograms from PET scans were reconstructed using a three-dimensional maximum a posteriori algorithm with scatter correction and CT-based correction for attenuation. PET and CT images were coregistered and analyzed using Inveon Research Workplace (Siemens). The mean % ID/g in different tissues was extracted by manually creating regions of interest (ROI) on fused PET/CT images. GraphPad Prism 9 (GraphPad Software, San Diego, CA, USA) was used for analyzing and plotting data.

## Figures and Tables

**Table 1:** Overview of the physicochemical properties, in vitro stability, and reactivity ^a^.

	[^64^Cu]Cu-NOTA-PEG_7_-H-Tz (1)	[^111^In]In-DOTA-PEG_11_-BisPy-Tz (2)
MW (g/mol)	1163.18	1386.31
Metal Complex Net Charge	−1	0
log*D*_7.4_	−2.44 ± 0.08^c^	−4.51 ± 0.07 ^b^
Rate constants (M^−1^ s^−1^) (in dioxane @ 25 °C)	216 ^d^	230 ^d^
Stability (%) -PBS (after 2h @ 37 °C)	87.0 ± 0.6 ^c^	96.7 ± 0.3 ^e^
Stability (%) -Serum (after 2h @ 37 °C)	84.7 ± 4.8 ^c^	86.8 ± 1.1 ^e^
Serum protein binding (%)	N.D.	5.6 ± 1.2 ^b^

^a^ Comparison of various physicochemical properties and in vitro stability where MW = molecular weight and log*D*_7.4_ = distribution coefficient at physiological pH (7.4). Second order rate constants are estimated from measurements of the Tz structural classes with *trans*-cyclooctence (TCO). Stability represents fraction of intact Tz. N.D = not determined; ^b^ data taken from reference [23], ^c^ data taken from reference [3], ^d^ data taken from reference [24], and ^e^ data taken from reference [4].

## Data Availability

The data presented in this study are available on request from the corresponding author.

## Sample Availability

Samples of NOTA-PEG_7_-H-Tz and DOTA-PEG_11_-BisPy-Tz are available from the authors.
